# The Fallacies of Popular Bacterial Research

**Published:** 1887-08

**Authors:** George W. Lewis

**Affiliations:** Buffalo, N. Y.


### THE FALLACIES OF POPULAR BACTERIAL RESEARCH.

By GEORGE W. LEWIS, Jr., A.M., M.D., Buffalo, N. Y.

I venture to say that every science has suffered more or less in its earlier development from the unprincipled and unguarded assertions of a certain well-defined class of men whose only prompter is an insatiate desire for notoriety, and whose only qualification that attracts attention to their sweeping statements is an established position in some other calling. I don't know that I should say "suffered from such assertions;" rather, perhaps, should it be, benefitted from them, for later on it is these very statements, far-reaching and groundless as they are, that serve by their contrast to strengthen and to disseminate the underlying principles of that science. It suffers only through the delay caused by the heaping on of so much trash and the consequent sifting that is rendered necessary.

The striking features, however, of these so-called "new discoveries" and empty criticisms are (1) that they are not advanced, as a rule, until their supporters realize that the subject has assumed enough importance to warrant the attraction of popular attention to their statements; and (2) that the promulgators of the class of work to which I refer are as profoundly ignorant of their subject as the most superficial knowledge of it can well make them. I take it that Pope's expression, "A little knowledge is a dangerous thing," was meant particularly for this class of individuals. Nevertheless, we all know that the more reckless and sweeping the assertion the more certain it is to attract widespread attention, and this is the only object its maker desires. It matters little to most of them how soon it is disproved, so long as timely notice is taken of it. It may be stated as a well-based induction that the more careful the investigator and the more complete his mastery over the endless practical difficulties which surround experimentation on his subject, the more certain are his experiments to give a guarded result; while extravagant and unfounded assertions are no less sure to crown the efforts of the unskilled.

These general remarks are intended as a preface to the subject in hand, for I don't believe that any science has ever suffered more for the cause of notoriety than has bacteriology. The reason for this seems to lie largely in the fact that the science is being developed under very peculiar circumstances. In the first place, it is one of the few technical sciences in which the great mass of people takes deep interest. It is easy, therefore, for those possessed of only a superficial knowledge to play upon the popular mind. If no more harm were done, there would be little cause for writing. But taking advantage of the combined deep interest, yet profound ignorance, of the masses, there has sprung up a vast number of self-styled discoverers, whose efforts, instituted solely for advertising purposes, being little less than ridicule upon the germ theory of disease. Private laboratories, fitted up with costly apparatus, are made to stand for extensive experimental research on the part of the owners, and, in their own immediate circles at least, no opportunity is lost to impress others with the idea that they are more or less closely identified with the onward progress of the science. Another cause, which undoubtedly militates against the speedy uprootal of this sort of charlatanism, is the impetus given it by the outbreak of an epidemic. Here a deal of notoriety is gained at the expense of popular fear.

Scarce, indeed, are the issues of even our most representative medical journals in which more or less space is not given up to "new discoveries" in bacteriology, always, of course, associating the name of Dr. So-and-so as the discoverer. Occasionally a few unimportant details relative to the peculiar circumstances under which the discovery was made, and calculated solely to enlist the popular mind, are given. Very seldom is mention made of culture or inoculation experiments, but great stress is always laid upon the microscopic appearances of the organism, and the easy or difficult manner in which it takes the stain, and perhaps other comparatively worthless data. More often, however, we are simply informed that the discovery has been made, and unfortified by details that would assist subsequent observers in confirming the experiments, all else is left as the work of a prolific imagination.

Now and then the monotony of "new discovery" trash (this word in lieu of a better one) is varied by a vigorous and wordy attack upon some demonstrated and generally-accepted organism. It seems unnecessary to add that the success or failure of such attacks depends largely, so far as the desired notoriety is concerned, upon the ability of the ones who make them to replace the blighted (?) remains of the old theory with a "new discovery." Sometimes the devices resorted to and the claims made are to say the least unique. A fair sample of the indefiniteness which invariably characterizes such work is seen in the following, which appeared in the editorial columns of the *New York Medical Record* of February 26, 1887, and upon which the editor passed a very just criticism.

The article has reference to the report of the cholera commission dispatched to Spain last year by the combined action of the Royal Society, the University of Cambridge, and the Association for the Promotion of Scientific Research. The commission consisted of Drs. Roy, Graham, Brown and Sherrington, and a review of their report is thus given by the journal named: "Twenty-five typical cases of cholera were examined either immediately or at a short interval after death, with the result that Koch's comma bacillus was not discovered in the intestinal canal in all the cases. In some this microbe was present in great abundance; in others it was far less conspicuous; while in many undoubted cases, where death occurred before the re-action stage set in, it could not be detected at all. These observations were confirmed by the results of plate cultivations in gelatine and agar-agar. Moreover, it was found that, when present, the comma bacilli were collected either on the surface of the mucosa or so close to it as to suggest a penetration of the epithelium after death, but in the majority the organism could not be found in the mucous membrane, or in any of the tissues and organs. These results, which are directly opposed to Koch's, are considered to be conclusive against the bacillus, having a pathogenetic relation to the disease, but it is suggested to be the cause of the premonitory diarrhœa, which is held not to be a mild attack of Asiatic cholera, but only a predisposing condition. Having satisfied themselves that the comma bacillus is not the cause of cholera, these investigations similarly dismiss the claims of Emmerich's straight bacillus to that distinction, and also state that they were unable to recognize Klein's straight bacillus in any of their preparations."

I have also read a copy of the original report, and the above is as concise a review of it as can be given. Here we have a good illustration of the broad assertions unsustained by reliable evidence to which I have referred in the first part of this paper. The report is based upon an examination of only twenty-five cases. Compare this with Koch's three years of uninterrupted study in cholera-infected localities before a single utterance was given to the world. The commission is frank to acknowledge that its report is somewhat premature, further investigation being needed, especially in the line of artificial cultivation. Its hasty dismissal of Koch's theory is unwarranted and savors strongly of English prejudice. Its reasoning, too, shows a lack of familiarity with Koch's views. Now listen to the indefinite character of what might naturally be expected to follow: "After much research another fungus was discovered which is believed to be pathogenetic. It consists of granular masses and a delicate mycelium, which could not be stained without difficulty, and was pronounced by Messrs. Vine and Gardiner to belong to the crytridiaceæ, a class which includes many rapidly-growing and virulent parasites of vegetables. The difficulties of its detection may have led to its being overlooked by former observers, while the objection of possible after-contamination is met and refuted."

The criticism offered by the editor is as follows: "It is, we venture to say, highly improbable that the researches above described will have much weight against the long-continued and careful observations of a trained mycologist like Koch."

The societies represented by these four men are the leading organizations of Great Britain in scientific research, but I doubt if much reliance is placed by them in the report of this commission. Their proceedings are usually both definite and trustworthy, but here both characteristics are lacking. Is there a single sentence in the whole report that conveys the slightest clue to assist subsequent observers in confirming their views? Not one that I can detect. They say the new fungus consists of granular masses and a delicate mycelium which cannot be stained without difficulty. They even go so far as to classify the new fungus. Now, what fungus, pray, does not consist of granular masses and a more or less delicate mycelium? These are the two known characteristics of all fungi. With regard to the difficult staining, I shall have occasion later, in speaking of the tubercle organism, to refer to its unreliability as a primary factor in diagnosis. It is valuable only as a confirmatory measure. Moreover, it is not by any means a constant quality even in the same organism. Subject, for example, several cultures of a given bacterium to different conditions of temperature and nourishment, each one maintained at the same throughout its growth, and you will be sure to notice degrees of stain-taking. This is, perhaps, less noticeable in the tubercle bacillus, on account of its limits of temperature being more closely defined.

The position which bacteriology occupies to-day in its relation to health and disease has been gained solely through perfected methods of studying minutely the life histories of the various organisms known to us, and yet the implicit confidence which is placed by many in microscopic appearances as a basis of diagnosis in bacterial affections clearly indicates the utter unreliability of a large proportion of the work done in this field. For the purpose of ascertaining beyond all doubt whether a micro-organism is actually the *causa causaus* of a disease, Koch has laid down the following postulates, which are strictly adhered to by all careful workers: (*a*) The micro-organism must be found in the blood, lymph or diseased tissues of man, or animal, suffering from, or dead of, the disease; (*b*) the micro-organisms must be isolated from the blood, lymph or tissues, and cultivated in suitable media outside of the animal body—these pure cultivations must be carried on through successive generations of the organism; (*c*) a pure cultivation thus obtained must, when introduced into the body of a healthy animal, produce the disease in question; (*d*) lastly, in the inoculated animal the same micro-organism must again be found. These steps naturally suggest a sequence in the various processes which must be adopted in a practical study of micro-organisms associated with disease.

Notwithstanding the axiomatic character of these postulates, and, in reality, the short time required for the execution of the necessary steps in each, we are every now and then led to believe, from articles in our journals, that more speedy and quite as trustworthy diagnoses can be made from microscopic examinations of the discharges peculiar to the diseases in question. This is particularly the case with tuberculosis. The methods of treating the sputum previous to the examination are both varied and numerous, each having its devotees; but they all unite in a dependence upon difficult stain-taking as the one strong point in the diagnosis. Biedert, whose plan, as given in the *Medical Record* of March 19th, is perhaps the latest, claims that the smallest possible number of bacilli, if present, can be detected. The essential features of his method are as follows: "A tablespoonful of the suspected sputa is added to twice that quantity of water and fifteen drops of a strong solution of soda. This mixture is boiled until it becomes quite fluid, and then diluted with about two ounces more of water. After being again boiled, the mixture is nearly homogeneous, and free from lumps and particles. If, on cooling, a thin fluid does not result, more water may be added. A conical vessel now receives the mixture, and after two or three days the supernatant fluid, having deposited its bacilli, can be decanted. The sediment, after energetic stirring, can now be examined in the ordinary manner." This method is open to additional objection on account of the dangers of after-contamination.

From the comparatively healthy mouth there have been isolated various micro-organisms that, so far as known, have no pathological significance. Any uncleanly condition, arising from laxity in the use of the brush or tooth-pick, whereby animal and vegetable matters are exposed to the process of decomposition, greatly increases the number and character of these organized bodies. The saliva, too, teems with living creatures, which, like the above, under the microscope bear a close resemblance to each other. How, then, I ask, can a trustworthy diagnosis be made from microscopic examinations of sputum, which contains, besides the possible tubercle bacilli, numerous other bacteria of like form and appearance? It will, no doubt, be claimed that a sufficient distinction can be arrived at through the staining peculiarities of the tubercle organism. Here, however, I would say that at least two of the non-pathogenic forms from the human mouth (Weinhauer's Bacillus and Babe's Bacillus), with which the writer is acquainted, can only be stained successfully by adopting some one of the methods recommended for the tubercle bacillus. In fact, several of the species found in the mouth were not discovered till late, on account of the difficulties experienced in staining them. Reference has been made, moreover, to the inconstancy of this quality of stain-taking, owing to the influence of different conditions of temperature and nourishment upon their organization. If the tubercle bacillus possessed distinctive microscopic characteristics, like the Anthrax Bacillus for example, there would be cause for placing some reliance in its appearance, but, unfortunately, it has no characteristic under the microscope that is not shared equally by other bacteria in the field of vision.

If the discovery of the tubercle bacillus is to have any real value from a therapeutic standpoint, it is essential that it be detected in the incipient stage of the disease. To do this requires not only a very exact primary method, but all the available checks that can possibly be interposed in the various steps necessary for its confirmation. The one respect in which all micro-organisms differ is their manner of growth. Here, then, is manifestly the starting point from which every reliable diagnosis of bacterial disease must proceed. Other measures of whatsoever sort are to be regarded as subservient to this, and to be of value only so far as they confirm the steps already taken.

Before closing this paper, I wish to speak of the importance of maintaining an even temperature throughout the growth of the organism. It is a feature in bacterial study too often neglected, and one which, if allowed to pass unheeded through successive generations, is certain to result in more or less pronounced modifications as regards structure and development. It is a question, too, whether intentional neglect in this respect will not, in time, check, or even destroy permanently, the pathogenetic tendencies of many of the bacteria. Every organized body has its normal limits of temperature, beyond which it cannot pass without suffering. It is a factor in their well-being next in importance to suitable nourishment. There is little doubt that these unicellular bodies are quite as sensitive to changes in temperature as are the most complicated and highly-developed of the vegetable kingdom.

